# Exploring xylose metabolism in *Spathaspora* species: *XYL1.2* from *Spathaspora passalidarum* as the key for efficient anaerobic xylose fermentation in metabolic engineered *Saccharomyces cerevisiae*

**DOI:** 10.1186/s13068-016-0570-6

**Published:** 2016-08-05

**Authors:** Raquel M. Cadete, Alejandro M. de las Heras, Anders G. Sandström, Carla Ferreira, Francisco Gírio, Marie-Françoise Gorwa-Grauslund, Carlos A. Rosa, César Fonseca

**Affiliations:** 1 Departamento de Microbiologia, ICB, C.P. 486, Universidade Federal de Minas Gerais, Belo Horizonte, Minas Gerais 31270-901 Brazil; 2 Laboratório Nacional de Energia e Geologia, I.P., Unidade de Bioenergia, Estrada do Paço do Lumiar 22, 1649-038 Lisbon, Portugal; 3 Department of Applied Microbiology, Lund University, PO Box 124, 22100 Lund, Sweden; 4 Novozymes A/S, Krogshøjvej 36, 2880 Bagsværd, Denmark; 5 Section for Sustainable Biotechnology, Aalborg University Copenhagen, A. C. Meyers Vænge 15, 2450 Copenhagen SV, Denmark

**Keywords:** *Spathaspora* species, *Spathaspora passalidarum*, *Saccharomyces cerevisiae*, Xylose fermentation, NADH-preferring xylose reductase, Bioethanol, *XYL1.2*

## Abstract

**Background:**

The production of ethanol and other fuels and chemicals from lignocellulosic materials is dependent of efficient xylose conversion. Xylose fermentation capacity in yeasts is usually linked to xylose reductase (XR) accepting NADH as cofactor. The XR from *Scheffersomycesstipitis*, which is able to use NADH as cofactor but still prefers NADPH, has been used to generate recombinant xylose-fermenting *Saccharomyces cerevisiae*. Novel xylose-fermenting yeasts species, as those from the *Spathaspora* clade, have been described and are potential sources of novel genes to improve xylose fermentation in *S. cerevisiae*.

**Results:**

Xylose fermentation by six strains from different *Spathaspora* species isolated in Brazil, plus the *Sp. passalidarum* type strain (CBS 10155^T^), was characterized under two oxygen-limited conditions. The best xylose-fermenting strains belong to the *Sp. passalidarum* species, and their highest ethanol titers, yields, and productivities were correlated to higher XR activity with NADH than with NADPH. Among the different *Spathaspora* species, *Sp. passalidarum* appears to be the sole harboring two XYL1 genes: *XYL1.1*, similar to the *XYL1* found in other *Spathaspora* and yeast species and *XYL1.2*, with relatively higher expression level. XYL1.1p and XYL1.2p from *Sp. passalidarum* were expressed in *S. cerevisiae* TMB 3044 and XYL1.1p was confirmed to be strictly NADPH-dependent, while XYL1.2p to use both NADPH and NADH, with higher activity with the later. Recombinant *S. cerevisiae* strains expressing XYL1.1p did not show anaerobic growth in xylose medium. Under anaerobic xylose fermentation, *S. cerevisiae* TMB 3504, which expresses XYL1.2p from *Sp. passalidarum*, revealed significant higher ethanol yield and productivity than *S. cerevisiae* TMB 3422, which harbors XYL1p N272D from *Sc. stipitis* in the same isogenic background (0.40 vs 0.34 g g_CDW_^−1^ and 0.33 vs 0.18 g g_CDW_^−1^ h^−1^, respectively).

**Conclusion:**

This work explored a new clade of xylose-fermenting yeasts (*Spathaspora* species) towards the engineering of *S. cerevisiae* for improved xylose fermentation. The new *S. cerevisiae* TMB 3504 displays higher XR activity with NADH than with NADPH, with consequent improved ethanol yield and productivity and low xylitol production. This meaningful advance in anaerobic xylose fermentation by recombinant *S. cerevisiae* (using the XR/XDH pathway) paves the way for the development of novel industrial pentose-fermenting strains.

**Electronic supplementary material:**

The online version of this article (doi:10.1186/s13068-016-0570-6) contains supplementary material, which is available to authorized users.

## Background

The lignocellulosic ethanol technology is reaching the commercial scale [1] and is enabling the industrial production of other bio-based products from lignocellulosic materials. Lignocellulose is the major structural component of plants and consists of cellulose, hemicellulose and lignin [2]. Lignocellulosic substrates are the largest source of fermentable sugars for the production of fuels and chemicals and the economic viability of second generation (2G) or lignocellulosic ethanol technology is dependent on the complete and efficient conversion of the carbohydrates, including those from the cellulosic and hemicellulosic fractions, primarily glucose and xylose [3, 4].

One of the key challenges for cost-effective lignocellulosic ethanol is the availability of robust microorganisms able to efficiently ferment all sugars present in lignocellulosic hydrolysates [1]. To address this bottleneck, *Saccharomyces cerevisiae*, the most commonly microorganism used in industrial alcoholic fermentations, has been engineered towards efficient xylose fermentation capacity [1], since other microorganisms, including native xylose-fermenting yeasts, can hardly convert xylose into ethanol at high titers, yields or productivities under industrially relevant ethanol production conditions, such as strict anaerobic environment, high osmotic stress, or high concentration of ethanol and inhibitors present in lignocellulosic hydrolysates [5]. Two metabolic engineering approaches have been followed to confer *S. cerevisiae* the ability to ferment xylose: the heterologous expression of xylose isomerase (XI) from bacteria or anaerobic fungi, which catalyzes the direct isomerization of d-xylose to d-xylulose [6–8]; and the heterologous expression of xylose reductase (XR) and xylitol dehydrogenase (XDH) from native xylose-fermenting yeasts (e.g., *Scheffersomyces stipitis*), which convert d-xylose into xylitol and xylitol into d-xylulose, respectively [7, 9]. The XR from *Sc. stipitis* uses both NADPH and NADH as cofactor, while XDH is NAD^+^-dependent enzyme [10]. Under anaerobiosis, engineered *S. cerevisiae* expressing XR/XDH from *Sc. stipitis* secrete significant amounts of xylitol due to scarcity of NAD^+^ [11], with a negative impact on the ethanol yield and productivity. To reduce xylitol production, enzyme engineering strategies have been applied to generate XR and/or XDH mutants with increased affinity for NADH and/or NADP^+^, respectively [12–16]. Among the modification induced or selected, the N272D mutation in *XYL1* from *Sc. stipitis* resulted in an increased preference for NADH in this yeast [14], and a recombinant *S. cerevisiae* expressing the same gene with N272D was able to grow anaerobically in xylose [15].

Conversely, microbial biodiversity has been a significant source of innovation in biotechnology [17]. Novel (non-conventional) yeasts from various habitats and some of their unique traits are source of biocatalysts for direct application in industrial processes or for metabolic engineering of industrial microorganisms, such as the yeast *S. cerevisiae* [18, 19]. Following extensive investigation on *Schefersomyces stipitis* and other xylose-fermenting strains, *Spathasporapassalidarum* was revealed as a novel yeast species capable of efficient xylose fermentation to ethanol [20–22]. More recently, five new species from the *Spathaspora* clade were isolated from Brazilian ecosystems [23, 24]. In addition, novel strains of *Sp. passalidarum* were obtained in Brazil [25]. In this context, the present work aimed to explore the xylose metabolism in these novel Brazilian *Spathaspora* strains through physiological, biochemical, and molecular characterization and further metabolic engineering of *S. cerevisiae* to assess the potential on novel traits towards superior xylose fermentation into ethanol under anaerobic conditions.

## Results

### Xylose fermentation by *Spathaspora* species under two different oxygen-limited conditions

Six yeasts strains isolated from Brazilian habitats, *Sp. arborariae* UFMG-CM-Y352^T^, *Sp. brasiliensis* UFMG-CM-Y353^T^, *Sp. passalidarum* UFMG-CM-Y469, *Sp. roraimanensis* UFMG-CM-Y477^T^, *Sp. suhii* UFMG-CM-Y475^T^, and *Sp. xylofermentans* UFMG-CM-Y478^T^, plus the reference strain *Sp. passalidarum* CBS 10155^T^, were studied under two different oxygen-limited conditions corresponding to an oxygen transfer rate (OTR) of approximately 1–2 mmol L^−1^ min^−1^ (severe) and 10–15 mmol L^−1^ min^−1^ (moderate). The results of the fermentation parameters including ethanol and xylitol titers (g L^−1^) yields (g g^−1^) are summarized in Table 1 (Figures in Additional file 1). All yeasts were able to consume xylose under both oxygen-limited conditions, but the specific xylose consumption rates were higher under severe oxygen-limited conditions for both *Sp. passalidarum* strains, higher under moderate oxygen-limited conditions for *Sp. brasiliensis* and *Sp. suhii*, and similar under both conditions tested for *Sp. arborariae*, *Sp. roraimanensis*, and *Sp. xylofermentans*. Regardless the oxygen-limited condition tested, the majority of the yeasts showed xylose consumption over 99 %, except *Sp. xylofermentans* (55.4 %) and *Sp. brasiliensis* (91.5 %) under moderate oxygen-limited conditions, and *Sp. suhii* (14.8 %), *Sp. brasiliensis* (31.5 %), and *Sp. xylofermentans* (97.5 %) under severe oxygen-limited conditions. Both *Sp. passalidarum* strains showed a remarkable high volumetric xylose consumption rate under both oxygen-limited conditions tested, with a virtually complete consumption between 18 h and 24 h. Still, under moderate oxygen-limited conditions, *Sp. roraimanensis* and *Sp. suhii* revealed similar specific xylose consumption rate, as less biomass is produced by these strains. Under severe oxygen-limited conditions, *Sp. passalidarum* presents superior specific xylose consumption rate.

**Table 1:** d-Xylose consumption and product formation (biomass, ethanol, and xylitol) (g L^−1^) in YPX fermentation assays (d-xylose, 40–50 g L^−1^) with *Spathaspora* species under moderate and severe oxygen-limited conditions, respectively

Oxygen-limited conditions	Yeast species	Strain number	Time of maximum ethanol production (h)	d-xylose consumption (%)	*r*_s_ (g g_CDW_^−1^ h^−1^)	Biomass (g_CDW_ L^−1^)	*Y*_x/s_ (g g^−1^)	Ethanol (g L^−1^)	$Y_{{{\text{p}}/{\text{s}}}}^{\text{et}}$ (g g^−1^)	*r*_et_ (g g_CDW_^−1^ h^−1^)	Xylitol (g L^−1^)	*Y*_p/s_^xy^ (g g^−1^)
Moderate	*Spathaspora arborariae*	UFMG-CM-Y352^T^	72	99.2	0.28	12.1 ± 0.6	0.24	10.6 ± 0.1	0.21	0.12	6.4 ± 0.1	0.13
	*Sp. brasiliensis*	UFMG-CM-Y353^T^	60	91.5	0.29	8.3 ± 0.6	0.18	3.7 ± 0.5	0.08	0.03	22.9 ± 0.2	0.46
	*Sp. passalidarum*	UFMG-CM-Y469	18	99.3	0.45	8.3 ± 0.7	0.19	20.2 ± 0.1	0.47	0.22	1.1 ± 0.0	0.03
	*Sp.* *roraimanensis*	UFMG-CM-Y477^T^	48	100	0.43	4.8 ± 0.1	0.09	5.8 ± 0.3	0.11	0.07	22.7 ± 0.3	0.43
	*Sp. suhii*	UFMG-CM-Y475^T^	48	100	0.44	4.7 ± 0.2	0.10	5.9 ± 0.1	0.12	0.09	22.4 ± 0.2	0.46
	*Sp. xylofermentans*	UFMG-CM-Y478^T^	48	55.4	0.18	8.3 ± 0.8	0.31	2.6 ± 0.2	0.10	0.02	9.0 ± 0.8	0.33
	*Sp. passalidarum*	CBS 10155^T^	18	99.1	0.43	8.9 ± 0.2	0.21	20.3 ± 0.2	0.48	0.21	0.6 ± 0.0	0.02
Severe	*Sp. arborariae*	UFMG-CM-Y352^T^	120	99.3	0.32	5.5 ± 0.0	0.11	16.0 ± 0.72	0.32	0.11	9.0 ± 0.03	0.18
	*Sp. brasiliensis*	UFMG-CM-Y353^T^	72	31.5	0.19	3.2 ± 0.6	0.21	2.6 ± 0.82	0.16	0.09	11.9 ± 0.88	0.47
	*Sp. passalidarum*	UFMG-CM-Y469	24	99.4	0.69	4.1 ± 0.1	0.10	20.5 ± 0.6	0.48	0.30	1.4 ± 0.01	0.03
	*Sp.* *roraimanensis*	UFMG-CM-Y477^T^	144	100	0.39	5.1 ± 0.6	0.11	3.0 ± 0.1	0.06	0.02	27.4 ± 0.4	0.56
	*Sp. suhii*	UFMG-CM-Y475^T^	–	14.8	0.16	0.3 ± 0.1	0.04	–	–	–	6.4 ± 0.6	0.92
	*Sp. xylofermentans*	UFMG-CM-Y478^T^	144	97.5	0.18	6.9 ± 0.7	0.15	3.7 ± 0.4	0.08	0.02	24.4 ± 1.3	0.51
	*Sp. passalidarum*	CBS 10155^T^	24	99.4	0.57	4.5 ± 0.2	0.11	20.5 ± 0.1	0.48	0.28	1.0 ± 0.2	0.02

*r*_*s*_ specific xylose consumption rate, *r*_*et*_ specific ethanol production rate, *Y*_*x/s*_ biomass yield; $Y_{p/s}^{et}$ ethanol yield, $Y_{p/s}^{xy}$ xylitol yield

Ethanol and/or xylitol were the major products from xylose metabolism. Accordingly, yeasts were grouped as: ethanol producers, comprising species showing ethanol as main product, *Sp. passalidarum* strains and *Sp. arborariae*; and xylitol producers, corresponding to *Sp. brasiliensis*, *Sp. roraimanensis*, *Sp. suhii* and *Sp. xylofermentans*. Among xylitol producers, *Sp. roraimanensis* and *Sp. xylofermentans* showed higher xylitol concentration under severe oxygen-limited conditions (27.4 and 24.4 g L^−1^, respectively), whereas *Sp. brasiliensis*, *Sp. roraimanensis* and *Sp. suhii* achieved the highest xylitol titers under moderate oxygen-limited conditions (22.9, 22.7, and 22.4 g L^−1^, respectively). Lower xylitol production observed for *Sp. brasiliensis* and *Sp. suhii* (11.9 and 6.4 g L^−1^, respectively) under severe oxygen-limited conditions is associated with low xylose consumption rates and incomplete xylose metabolism. All xylitol producers revealed low ethanol yields, between 0.06 and 0.16 g g^−1^. *Spathaspora suhii* was the sole species unable to produce ethanol under severe oxygen-limited conditions, as xylose consumption and biomass formation were very low. *Spathaspora passalidarum,* an ethanol producer, achieved the highest ethanol titers (over 20 g L^−1^), yields (0.47–0.48 g g^−1^), and productivities (0.21–0.30 g g_CDW_^−1^ h). The specific ethanol productivity was approximately 30 % superior under severe oxygen-limited conditions. In addition, *Sp. passalidarum* produced the lowest xylitol concentrations (0.6 to 1.4 g L^−1^) corresponding to yields below 0.04 g g^−1^. *Spathaspora arborariae* achieved ethanol titers of 10.6 and 16.0 g L^−1^ under moderate and severe oxygen limitation, respectively, but ethanol productivities were much lower than those found with *Sp. passalidarum* (0.11–0.12 g g_CDW_^−1^ h^−1^). Although this yeast generated ethanol as main product, a considerable amount of xylitol was obtained, around 6.4 and 9.0 g L^−1^ under moderate and severe oxygen-limited conditions, respectively.

### Xylose reductase and xylitol dehydrogenase activities in *Spathaspora* species

The enzyme activities associated with the first steps of xylose metabolism, xylose reductase (XR), and xylitol dehydrogenase (XDH) were determined in *Spathaspora* species cell extracts after 16 h xylose metabolism under moderate or severe oxygen-limited conditions (Table 2). All the yeasts showed xylitol dehydrogenase activity strictly dependent of NAD^+^. In turn, xylose reductase activity was NADPH-dependent or accepted both NADH and NADPH depending on the groups described above. While xylitol producers (*Sp. brasiliensis*, *Sp. roraimanensis*, *Sp. suhii*, and *Sp. xylofermentans*) had strictly NADPH-dependent XR activities, the ethanol producers (*Sp. passalidarum* strains and *Sp. arborariae*) showed XR activities both using NADH and NADPH as cofactor. However, *Sp. arborariae* shown higher XR activity with NADPH, while both *Sp. passalidarum* strains revealed higher XR activity with NADH.

**Table 2:** Xylose reductase (XR) and xylitol dehydrogenase (XDH) activities in *Spathaspora* species, expressed in units (U) per mg protein [U (mg protein)^−1^], after 16 h of fermentation in YPX medium under moderate and severe oxygen-limited conditions, respectively

Oxygen-limited conditions	Yeast species	Strain number	XR			XDH
			NADH	NADPH	Ratio NADH/NADPH	NAD^+^
Moderate	*Spathaspora arborariae*	UFMG-CM-Y352^T^	0.54 ± 0.02	0.72 ± 0.06	0.75 ± 0.07	0.57 ± 0.05
	*Sp. brasiliensis*	UFMG-CM-Y353^T^	–	0.39 ± 0.02	–	0.18 ± 0.01
	*Sp. passalidarum*	UFMG-CM-Y469	0.83 ± 0.12	0.46 ± 0.06	1.81 ± 0.35	0.60 ± 0.07
	*Sp.* *roraimanensis*	UFMG-CM-Y477^T^	–	0.64 ± 0.04	–	0.74 ± 0.02
	*Sp. suhii*	UFMG-CM-Y475^T^	–	0.51 ± 0.03	–	0.74 ± 0.04
	*Sp. xylofermentans*	UFMG-CM-Y478^T^	–	0.09 ± 0.01	–	0.12 ± 0.01
	*Sp. passalidarum*	CBS 10155^T^	2.81 ± 0.31	1.62 ± 0.17	1.74 ± 0.26	1.80 ± 0.14
Severe	*Sp. arborariae*	UFMG-CM-Y352^T^	0.33 ± 0.04	0.61 ± 0.01	0.55 ± 0.07	0.84 ± 0.07
	*Sp. brasiliensis*	UFMG-CM-Y353^T^	–	0.35 ± 0.01	–	0.60 ± 0.01
	*Sp. passalidarum*	UFMG-CM-Y469	1.09 ± 0.12	0.63 ± 0.14	1.72 ± 0.43	1.17 ± 0.12
	*Sp.* *roraimanensis*	UFMG-CM-Y477^T^	–	0.26 ± 0.03	–	0.87 ± 0.01
	*Sp. suhii*	UFMG-CM-Y475^T^	–	0.20 ± 0.01	–	0.63 ± 0.04
	*Sp. xylofermentans*	UFMG-CM-Y478^T^	–	0.27 ± 0.01	–	0.54 ± 0.01
	*Sp. passalidarum*	CBS 10155^T^	4.07 ± 0.55	2.46 ± 0.27	1.66 ± 0.29	2.19 ± 0.05

Overall, the XDH activities determined under severe oxygen-limited conditions were higher than under moderate oxygen-limited conditions, except for *Sp. suhii*. On contrary, higher NADPH-dependent XR activities were measured under moderate oxygen-limited conditions, except for both *Sp. passalidarum* strains and *Sp. xylofermentans*. XR activities in *Sp. arborariae* UFMG-CM-Y352 are apparently lower under severe oxygen-limited conditions either with NADH or with NADPH as cofactor, while in *Sp. passalidarum*, superior XR activities are found at higher oxygen limitation, regardless the cofactor considered. The NADH/NADPH ratios for XR activities are slightly lower in *Sp. passalidarum* and *Sp. arborariae* strains under severe oxygen-limited conditions. Moreover, XRs enzymes of *Sp. passalidarum* have a remarkable higher activity with NADH, reaching NADH/NADPH ratio higher than 1.6. Indeed, in all *Spathaspora* species studied in this work, *Sp. passalidarum* was the sole species presenting this behavior. The *Spathaspora* species showing strict NADPH-dependent XR activity revealed that XR activities (Table 2) mostly correlate with the xylose consumption rate (Table 1), thus suggesting that under the conditions, tested XR plays a relevant role on the control of the metabolic flux during xylose assimilation.

### *XYL1* gene(s) and encoded proteins in *Spathaspora* species

**Fig. 1:**
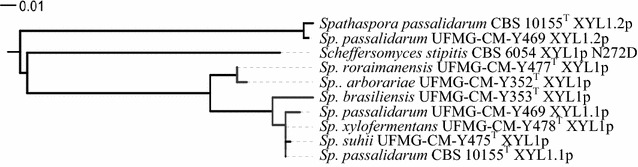
Phylogenetic relationship among *Spathaspora* species and *Scheffersomyces stipitis* (XYL1p N272D) based on XYL1p, XYL1.1p, and XYL1.2p amino acid sequences

Taking advantage of the available genome sequences from *Sp. passalidarum* CBS 10155^T^ (NRRL Y-27907^T^) [29] and *Sp. arborariae* UFMG-CM-Y352 (UFMG-HM-19.1A^T^, CBS 11463^T^) [30], a region of approx. 15 kb containing xylose reductase gene(s) (*XYL1*) from both strains were aligned and *XYL1* from *Sp. arborariae* UFMG-CM-Y352 revealed to be 89 % identical to *XYL1.1* from *Sp.passalidarum* CBS 10155^T^ (NRRL Y-27907^T^). A set of consensus primers able to amplify the complete coding sequences of both *SppaXYL1.1* and *SparXYL1* was designed (SpspXYL1.1_F and SpspXYL1.1_R). Moreover, a second *XYL1* was identified in *Sp.passalidarum* CBS 10155^T^ (NRRL Y-27907^T^) (*XYL1.2*), approximately 1 kb distant from *XYL1.1*, and is apparently absent in *Sp. arborariae* UFMG-CM-Y352. Therefore, a set of primers covering the complete *XYL1.2* of *Sp.passalidarum* (SppaXYL1.2_F and SppaXYL1.2_R) was also designed. The two sets of primers were used to identify *XYL1* in all *Spathaspora* species used in this study. *XYL1* was detected in all *Spathaspora* species (*XYL1.1* for *Sp. passalidarum* strains) (GenBank accession numbers: *Sp. arborariae*—KU170769; *Sp. brasiliensis*—KU170770; *Sp. xylofermentans*—KU170771; *Sp. suhii*—KU170772; *Sp. roraimanensis*—KU170773; *Sp. passalidarum* CBS 10155^T^—KU170774; and *Sp. passalidarum* UFMG-CM-Y469—KU170775), with a coding region of 957 base pairs (bp), resulting in XRs with 318 amino acids (aa). The existence of a second *XYL1* (*XYL1.2*) was only identified in *Sp. passalidarum* species (GenBank accession numbers: *Sp. passalidarum* CBS 10155^T^—KU170767 and *Sp. passalidarum* UFMG-CM-Y469—KU170768). *XYL1.2* coding regions have 954 bp, resulting in XRs with 317 aa. The nucleotide and amino acid sequences of *XYL1.2* from *Sp. passalidarum* CBS 10155^T^ and *Sp. passalidarum* UFMG-CM-Y469 display high identity, of 98 % and 99 %, respectively, with 15 bp and 3 aa difference. A phylogram was constructed based on the predicted amino acid sequences of the XRs among *Spathaspora* species (Fig. 1).

The comparison of *XYL1.1* and *XYL1.2*, and XYL1p and XYL1.2p within *Sp. passalidarum* strains showed that, besides the protein size difference in one amino acid, *Sp. passalidarum* CBS 10155^T^*XYL1.2* is 77 % identical to *XYL1.1*, and XYL1.2p is 74 % identical to XYL1.1p. Similarly, for *Sp. passalidarum* UFMG-CM-Y469, identities of 78 and 75 % among XYL1 genes and coded proteins, respectively, were observed. The phylogram (Fig. 1) reveals that *Sc. stipitis* XYL1p N272D is distant from both XYL1p/XYL1.1p and XYL1.2p of the *Spathaspora* clade.

### XYL1.1 and XYL1.2 transcript analysis in *Spathaspora passalidarum*

**Fig. 2:**
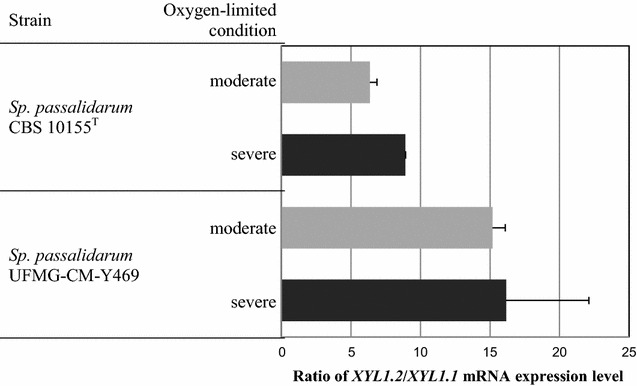
Ratio of *XYL1.2*/*XYL1.1* mRNA expression level in *Sp. passalidarum* CBS 10155^T^ and *Sp. passalidarum* UFMG-CM-Y469 after 16 h fermentation in YPX medium under moderate or severe oxygen-limited conditions. RT—real-time results analyzed through the Pfaffl method [40]

The levels of XYL1.1 and XYL1.2 transcripts were evaluated by real-time RT-PCR using actin (*SppaACT1*) and 18S ribosomal RNAs (*SppaRDN18*) as internal controls (Fig. 2). *XYL1.2* is more expressed than *XYL1.1* in both *Sp. passalidarum* strains, 6–9-folds in *Sp. passalidarum* CBS 10155^T^ and 15–16-folds in *Sp. passalidarum* UFMG-CM-Y469 under both oxygen-limited conditions. While *XYL1.2* expression is slightly lower under severe oxygen-limited conditions in *Sp. passalidarum* CBS 10155^T^, on contrary, in *Sp. passalidarum* UFMG-CM-Y469, its expression is slightly higher under severe than under moderate oxygen-limited conditions (data not shown).

### The effect of *SpXYL1.1* and *SpXYL1.2* expression in *Saccharomyces cerevisiae*

Within the *Spathaspora* species studied in this work, *Sp. passalidarum* is the sole presenting higher XR activities with NADH as cofactor and harboring two XYL1 genes, *XYL1.1* and *XYL1.2*. To study the influence of *XYL1.1* and *XYL1.2* from *Sp. passalidarum*, the genes were individually expressed in *S. cerevisiae* TMB 3044, the most suitable TMB strain for testing novel *XYL1* genes. The expression of the four genes in *S. cerevisiae* TMB 3044 generated two sets of *S. cerevisiae* strains: TMB 3501 and TMB 3502, expressing *XYL1.1* from *Sp. passalidarum* CBS 10155^T^ and UFMG-CM-Y469, respectively; TMB 3503 and TMB 3504, expressing *XYL1.2* from *Sp. passalidarum* CBS 10155^T^ and UFMG-CM-Y469, respectively. Prior to the transformation of *Sc. cerevisiae*, the absence of CUG codons in the *XYL1* genes has been confirmed.

The four recombinant *S. cerevisiae* strains were first tested under anaerobiosis in YNB-xylose medium. Since *S. cerevisiae* TMB 3501 and TMB 3502 were unable to grow anaerobically, the recombinant *S. cerevisiae* strains were characterized in terms of XR activity and cofactor specificity under aerobic conditions in YNB-xylose medium (Table 3). Strains *S. cerevisiae* TMB 3501 and TMB 3502 revealed strict NADPH-dependent XR activity, while *S. cerevisiae* TMB 3503 and TMB 3504 revealed dual cofactor utilization with higher activity when using NADH as cofactor (NADH/NADPH ratio of 1.34 and 1.21, respectively). However, the XR activity of *S. cerevisiae* TMB 3504 was approximately threefold the one determined in *S. cerevisiae* TMB 3503.

**Table 3:** Xylose reductase (XR) activity (200 mM xylose), expressed in units (U) per mg protein [U (mg protein)^−1^], of *S. cerevisiae* TMB 3501, TMB 3502, TMB 3503, and TMB 3504 after 48 h of aerobic cultivation in YNB-xylose medium

Yeast species	Strain number	XR		
		NADH	NADPH	Ratio NADH/NADPH
*S. cerevisiae*	TMB 3501	–	2.88 ± 0.12	–
*S. cerevisiae*	TMB 3502	–	2.38 ± 0.08	–
*S. cerevisiae*	TMB 3503	1.77 ± 0.13	1.32 ± 0.01	1.34 ± 0.10
*S. cerevisiae*	TMB 3504	4.97 ± 0.30	4.12 ± 0.36	1.21 ± 0.13

**Fig. 3:**
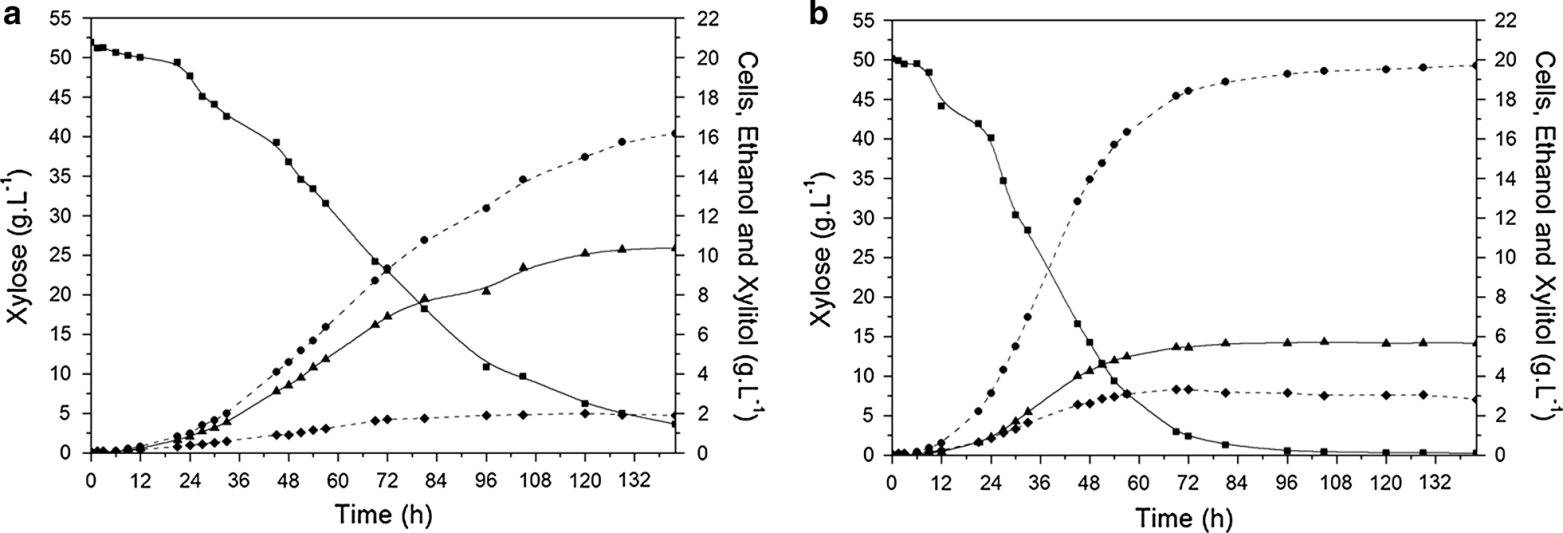
Time course of substrate consumption and product formation by *S. cerevisiae* strains TMB 3422 (**a**) and TMB 3504 (**b**) under anaerobic batch fermentation of 50 g L^−1^ xylose. *Square* (*continuous line*) xylose; *circle* (*dashed line*) ethanol; *triangle* (*continuous line*) xylitol; *diamond* (*dashed line*) cells

Based on these results, *S. cerevisiae* TMB 3504 was selected to be evaluated in anaerobic batch fermentation under controlled conditions with 50 g L^−1^ xylose. The strain *S. cerevisiae* TMB 3422, which carries the mutated *Sc. stipitis* XR (N272D) [14, 15], was used as benchmarking for TMB 3504 (Fig. 3; Table 4). *Saccharomyces cerevisiae* TMB 3504 showed more than 70 % of sugar conversion into ethanol in approximately 48 h (Fig. 3b) at relatively constant specific xylose consumption and ethanol production rates and with an ethanol yield of 0.40 g g^−1^. The ethanol titer slightly increases up to approximately 18 g L^−1^ at 72 h and 20 g L^−1^ at 142 h. Maximum specific growth rate (0.11 vs 0.07 h^−1^), specific substrate consumption (0.76 vs 0.58 g g_CDW_^−1^ h^−1^) rate, and specific ethanol productivity (0.33 vs 0.18 g g_CDW_^−1^ h^−1^) of *S. cerevisiae* TMB 3504 are significantly higher than those of *S. cerevisiae* TMB 3422 (Table 4), which in turn reveals a relatively constant ethanol production rate until approximately 100 h, reaching maximal ethanol concentration of approximately 16 g L^−1^ (Fig. 3a), i.e., an ethanol yield of 0.34 g g^−1^. The higher xylose consumption rate, and ethanol yield and productivity revealed by *S. cerevisiae* TMB 3504 when compared to *S. cerevisiae* TMB 3422 is linked lower xylitol yield (0.10 vs 0.21 g g^−1^) (Fig. 3; Table 4).

**Table 4:** D-xylose consumption, product formation (biomass, ethanol, and xylitol) and xylose reductase (XR) activity in anaerobic xylose fermentation (50 g L^−1^) with recombinant *S. cerevisiae* strains TMB 3422 and TMB 3504

Strains	*µ*_max_ (h^−1^)	*r*_s_ (g g_CDW_^−1^ h^−1^)	*Y*_x/s_ (g g^−1^)	$Y_{\text{p/s}}^{\text{et}}$ (g g^−1^)	*r*_et_ (g g_CDW_^−1^ h^−1^)	$Y_{\text{p/s}}^{\text{xy}}$ (g g^−1^)	XR		
							NADH	NADPH	Ratio NADH/NADPH
TMB 3422	0.07 ± 0.00	0.58 ± 0.10	0.04 ± 0.01	0.34 ± 0.01	0.18 ± 0.01	0.21 ± 0.01	0.67 ± 0.03	0.94 ± 0.02	0.71 ± 0.04
TMB 3504	0.11 ± 0.00	0.76 ± 0.06	0.06 ± 0.00	0.40 ± 0.00	0.33 ± 0.02	0.10 ± 0.02	1.13 ± 0.08	0.96 ± 0.01	1.18 ± 0.08

*µ*_*max*_ specific growth rate, *r*_*s*_ specific xylose consumption rate, *r*_*et*_ specific ethanol production rate, *Y*_*x/s*_ biomass yield; $Y_{p/s}^{et}$ ethanol yield; $Y_{p/s}^{xy}$ xylitol yield

XR activity was determined in crude cell-free extracts from *S. cerevisiae* TMB 3504 and TMB 3422 after approximately 48 h of anaerobic batch fermentation (Table 4). Both *S. cerevisiae* TMB 3504 and TMB 3422 showed NAD(P)H-dependent xylose reductase activities. However, whereas *S. cerevisiae* TMB 3422 displays higher XR activity with NADPH as cofactor (NADPH/NADH ratio of 0.71), *S. cerevisiae* TMB 3504 harbors an XR (*Sp. passalidarum* UFMG-CM-Y469 XYL1.2p) with higher activity when using NADH as cofactor (NADH/NADPH ratio of 1.18).

## Discussion

In the present study, a group of *Spathaspora* species, belonging to a relatively new clade of xylose-fermenting yeasts [20–26], has been studied in relation to their xylose-fermenting capacity and classified as xylitol or ethanol producers according to the major product of xylose metabolism. This physiological characterization was associated with the biochemical characterization of XR activity: the ethanol producers *Sp. passalidarum* and *Sp. arborariae* revealed XR activities with both NADH and NADPH as cofactors; the xylitol producers, *Sp. brasiliensis, Sp. roraimanensis, Sp. suhii*, and *Sp. xylofermentans,* showed XR activities strictly NADPH-dependent. As Crabtree-negative yeasts in the metabolism of xylose, ethanol production only takes place under oxygen-limited conditions. These conditions impair the efficient regeneration of NAD^+^ required for the xylitol oxidation by XDH, unless XR is able to use NADH as cofactor, thus liberating NAD^+^ for the next reaction catalyzed by XDH. The xylose fermentation capacity of yeasts has been directly correlated with the existence of an XR able to utilize NADH as cofactor [27]. Although, in this work, all the *Spathaspora* species tested were able to produce more than 2 g L^−1^ ethanol from xylose (40–50 g L^−1^), except *Sp. suhii* under severe oxygen-limited conditions, the requirement of an XR able to use NADH as cofactor for efficient xylose fermentation is confirmed.

Among the best wild xylose-fermenting yeasts described in the past decades, *Pachysolen tannophilus*, *Scheffersomycesshehatae* (including *Sc. lignosus* and *Sc*. *insectosa*) and *Scheffersomycesstipitis* stood out first, followed more recently by *Spathaspora* species [20–28]. The oxygen-limited conditions applied in this work confirmed *Sp. passalidarum* as the best xylose-fermenting yeast described so far, with ethanol yields close to the theoretical maximum (0.47–0.48 g g^−1^), a feature only achieved by *Sc. stipitis* and *Sc. lignosus* [25, 28]. However, *Sp. passalidarum* showed higher ethanol productivities than these yeasts. Still, as with other natural xylose-fermenting yeasts, the specific aeration requirements and the performance in hemicellulose hydrolysates [26] may difficult the utilization of *Sp. passalidarum* under industrial setup. The superior xylose fermentation behavior revealed by *Sp. passalidarum* was suggested to be related to the higher XR activity with NADH than with NADPH [21, 22]. This apparent preference for NADH in crude extracts, up to 1.8 of NADH/NADPH ratio [22], is confirmed in this work for both *Sp. passalidarum* strains tested, a trend that is not followed by the other *Spathaspora* species. The second major ethanol producer of this study *Sp. arborariae* revealed higher XR activity with NADPH, as other xylose-fermenting yeasts, such as *Sc. stipitis*. Although both *Sp. passalidarum* strains presented similar NADH/NADPH ratios for XR activity, this activity was 3.5-fold higher in *Sp. passalidarum* CBS 10155^T^.

The alignment of a genome region from the two species classified as ethanol producers, *Sp. passalidarum* [29] and *Sp. arborariae* [30], resulted in the identification of two genes coding xylose reductases in *Sp. passalidarum*, *XYL1.1*, and *XYL1.2*, separated by approximately 1 kb, but only one *XYL1* in *Sp. arborariae*. Consensus primers allowed the identification of complete (but apparently single) *XYL1* sequences in the remaining *Spathaspora* species. *XYL1.1* and *XYL1.2* from both *Sp. passalidarum* were individually expressed together with *Sc. stipitisXYL2* (coding a xylitol dehydrogenase) in *S. cerevisiae* TMB 3044 [7], a background with overexpressed pathway for xylose utilization and virtual absence of XR activity (Δ*gre3*). The resulting strains, *S. cerevisiae* TMB 3501 (*XYL1.1* from *Sp. passalidarum* CBS 10155^T^) and *S. cerevisiae* TMB 3502 (*XYL1.1* from *Sp. passalidarum* UFMG-CM-Y469), were unable to grow on minimal medium containing xylose as sole carbon and energy source under anaerobic conditions and, under aerobiosis, XR activity was, in both cases, strictly NADPH-dependent. On contrary, *S. cerevisiae* TMB 3503 (*XYL1.2* from *Sp. passalidarum* CBS 10155^T^) and TMB 3504 (*XYL1.2* from *Sp. passalidarum* UFMG-CM-Y469) were able to grow anaerobically in xylose medium, revealing XR activity with dual cofactor utilization with higher activity with NADH (NADH/NADPH ratio 1.2–1.3). Moreover, in *Sp. passalidarum*, *XYL1.2* is 6–16-folds more expressed than *XYL1.1* (6–9 for *Sp. passalidarum* CBS 10155^T^ and 15–16 for *Sp. passalidarum* UFMG-CM-Y469) under the (oxygen-limited) conditions tested for transcript analysis. This different expression levels between *XYL1.2* and *XYL1.1* may explain the overall higher XR activity with NADH found in crude extracts of *Sp. passalidarum* and the efficiency of this species on xylose fermentation.

The existence of an additional gene in *Sp. passalidarum* able to provide to this species a specific behavior with respect to D-xylose metabolism may be explained by the gene duplication phenomenon [31–34], evidenced by the *XYL1.1* and *XYL1.2* proximity (1 kb distance). Paralogous genes are rarely preserved in the genome and their maintenance is related to the differentiation and acquisition of a functional specialization or neofunctionalization [31, 32, 34]. The possible duplication of *XYL1* in *Sp. passalidarum* may have enabled an evolutionary adaptation and specialization of *XYL1.2* for oxygen-limited xylose utilization, which is much more expressed than *XYL1.1* under these conditions. In fact, the expression of duplicated genes tends to evolve asymmetrically, i.e., the expression of one copy evolved rapidly, while the other copy retains most of the expression profile found in the common ancestral gene [35].

The comparison of the XR coding genes as well as the predicted amino acid sequences in *Spathaspora* species allowed the identification of conserved and divergent regions. Percentages of identities between 89 and 100 % and 93 and 100 % were found by comparing, respectively, the sequences of *XYL1* and *XYL1.1*, and XYL1p and XYL1.1p among the *Spathaspora* species studied with the type strain of *Sp. passalidarum*. These results demonstrate a high degree of sequence conservation among the genes and proteins from the species analyzed. Interestingly, *XYL1* from *Sp. xylofermentans* shows 100 % identity with *XYL1.1* of *Sp. passalidarum* (type strain). Interestingly, XYL1p (XRs) can be grouped according to their cofactor utilization profile: (1) strictly NADPH-dependent, such as *XYL1* from *Sp. brasiliensis*, *Sp. roraimanensis*, *Sp. suhii*, and *Sp. xylofermentans*, and *XYL1.1* from *Sp. passalidarum*; (2) apparent preference for NADPH, such as *XYL1* from *Sc. stipitis* and *Sp. arborariae*; (3) apparent preference for NADH, *XYL1.2* from *Sp. passalidarum*. From the analysis of amino acid residues, 115 residues differ among groups, 44 are exclusively found in NADH-preferring XR sequences (XYL1.2p from *Sp. passalidarum*), and 1 in those able to use NADH (groups 2 and 3), which corresponds to a lysine (K) residue instead of an arginine (R) in position 59/60. Moreover, adjacent to the cofactor binding site of the enzyme, the highly conserved tetrad Ile-Pro-Lys-Ser (IPKS) [11, 33–35], in position 271/272, XYL1.2p from *Sp. passalidarum* display an aspartate (D) residue instead of asparagine (N) found in the remaining XYL1p [except that from *Sp. brasiliensis*, which displays a serine (S)] (Additional file 2). Protein engineering has been applied to modify cofactors preference of XRs [11–15]. Among the modification induced or selected, the N272D mutation in *XYL1* from *Sc. stipitis* resulted in an increased affinity for NADH in this yeast [13], and a recombinant *S. cerevisiae* expressing the same gene with N272D was able to grow anaerobically in xylose [14, 15].

This strain, *S. cerevisiae* TMB 3422 [14], was used as benchmarking for the characterization of the newly generated *S. cerevisiae* TMB 3504 (expressing XYL1.2p from *Sp. passalidarum* UFMG-CM-Y469) in anaerobic xylose fermentation, as these strains have an isogenic background. The *S. cerevisiae* TMB 3504 strain was able to efficiently convert d-xylose into ethanol with high yield and productivity and low xylitol accumulation. Compared to *S. cerevisiae* TMB 3422, the recombinant xylose-fermenting *S. cerevisiae* considered by Runquist et al. [15] as responsible for one of the highest ethanol yields already reported for engineering *S. cerevisiae* harboring the XR/XDH pathway, *S. cerevisiae* TMB 3504 displays approximately more 20 % ethanol yield (0.40 vs. 0.34 g g^−1^), less than 50 % xylitol yield (0.10 vs. 0.21 g g^−1^), and 80 % higher specific ethanol productivity, corresponding to a maximum ethanol titer of approximately 18 g L^−1^ after 72 h (from approx. 50 g L^−1^ xylose), almost half of the fermentation time observed for *S. cerevisiae* TMB 3422. This significantly higher volumetric productivity found for *S. cerevisiae* TMB 3504 is, in part, explained by the higher biomass yield (0.06 vs. 0.04 g g^−1^ in *S. cerevisiae* TMB 3422).

The major bottlenecks in recombinant xylose-fermenting *S. cerevisiae* are low ethanol productivity from xylose [15], poor xylose fermentation in the presence of inhibitors [36], poor xylose co-consumption with glucose [37], and, in those expressing the XR/XDH pathway, the considerable xylitol accumulation and consequent limited ethanol yield [7, 38]. To avoid xylitol accumulation, the majority of industrial xylose-fermenting strains have been constructed using the XI pathway [36], but the novel metabolic engineered laboratory *S. cerevisiae* strain (TMB 3504), harboring an XR with apparent preference for NADH, showed much lower xylitol accumulation together with a higher ethanol yield and productivity when compared to other recombinant XR/XDH xylose-fermenting *S. cerevisiae* strains [15].

## Conclusions

This work identifies crucial traits for xylose fermentation in *Spathaspora* genus, through physiological, biochemical, and molecular characterization and further functional expression of XRs in *S. cerevisiae*. The characterization of xylose fermentation, XR/XDH activities, and the identification and characterization of the XR coding genes (*XYL1*) allowed the selection of two genes, one from each *Sp. passalidarum* studied, coding for XR activity with apparent preference for NADH. *Saccharomyces cerevisiae* TMB3504 is a new recombinant xylose-fermenting strain harboring *XYL1.2* from *Sp. passalidarum* UFMG-CM-Y469. This strain revealed an ethanol yield of 0.40 g g^−1^ with specific productivity of 0.33 g g_CDW_^−1^ h^−1^ against 0.34 g g^−1^ and 0.18 g L^−1^ h^−1^ found with the reference strain used (*S. cerevisiae* TMB3422), previously reported as one of the best recombinant xylose-fermenting lab strain harboring an heterologous XR/XDH pathway (protein engineered XR from *Sc. stipitis*).

The rational bioprospective approach undertaken revealed *XYL1.2* from *Sp. passalidarum* as a key for efficient xylose anaerobic fermentation by *S. cerevisiae* when expressing the XR/XDH pathway. The results obtained with the novel laboratory xylose-fermenting *S. cerevisiae* strain bring new insights to the XR/XDH pathway as an alternative for industrial strain development towards the effective deployment of lignocellulosic ethanol.

## Methods

### Strains and maintenance

*Spathaspora. arborariae* UFMG-CM-Y352^T^ (=UFMG-HM-19.1A^T^ = CBS 11463^T^), *Sp. brasiliensis* UFMG-CM-Y353^T^ (=UFMG-HMD-19.3^T^ = CBS 12679^T^), *Sp. passalidarum* UFMG-CM-Y469 (=UFMG-HMD-1.1), *Sp. roraimanensis* UFMG-CM-Y477^T^ (=UFMG-XMD-23.2^T^ = CBS 12681^T^), *Sp. suhii* UFMG-CM-Y475^T^ (=UFMG-XMD-16.2^T^ = CBS 12680^T^), and *Sp. xylofermentans* UFMG-CM-Y478^T^ (UFMG-HMD-23.3^T^ = CBS 12682^T^) were obtained from the Coleção de Micro-organismos e Células da Universidade Federal de Minas Gerais—UFMG (Collection of Microorganisms and Cells of Universidade Federal of Minas Gerais), Belo Horizonte, Brazil. *Spathaspora arborariae* was isolated from rotting wood samples collected in an Atlantic Rain Forest and a Cerrado ecosystem in Brazil [23]. The other Brazilian *Spathaspora* strains were isolated from the same substrate in the Amazonian Forest, as described by Cadete et al. [24]. *Spathaspora passalidarum* CBS 10155^T^ was bought from the Centraalbureau voor Schimmelcultures (CBS) culture collection. *Saccharomyces cerevisiae* strains TMB 3044 and TMB 3422 were provided by the Teknisk Mikrobiologi (TMB) culture collection (Lund, Sweden). All strains were stored in 15 % glycerol at −80 °C.

### Xylose fermentations under oxygen-limited conditions with *Spathaspora* species

Fermentation experiments were carried out on YPX medium (yeast extract, 10 g L^−1^; peptone, 20 g L^−1^; d-xylose, 40–50 g L^−1^) in shake flasks under the pre-established oxygen-limited conditions, being (1) moderate: 100 mL working volume in 250 mL cotton plugged flask and (2) severe: 100 mL working volume in 100 mL rubber plugged flask with needle to allow the release of CO_2_. Oxygen transfer rate (OTR) was determined by direct measurements in the culture media (with or without cells), at several time intervals, with an oxygen electrode after sparging with nitrogen. Yeasts were pre-grown on YMA medium (glucose, 10 g L^−1^; peptone, 5 g L^−1^; yeast extract, 3 g L^−1^; malt extract, 3 g L^−1^; agar, 20 g L^−1^) for 24–48 h, and single colonies were transferred to 50 mL YPX in 250 mL shake flasks at 30 °C and 200 rpm. Cells were recovered by centrifugation at 2600*g* for 20 min, washed twice with sterile water, and suspended in the fermentation media as inoculum with a final concentration of 0.5 g_CDW_ L^−1^ [25]. The flasks were incubated at 30 °C and 200 rpm, and the fermentation was monitored by taking samples between 0 and 144 h, according to the conditions of oxygen limitation employed. Samples were stored at −20 °C until analysis. All experiments were performed in duplicate. Cell biomass was determined by correlating optical density (OD) at 600 nm (OD_600_) in a Thermo Spectronic Genesys 20 Model 4001/4 spectrophotometer (Thermo Scientific, Waltham, USA) with the cell dry weight (CDW), by means of a calibration curve previously constructed for each yeast strain. Xylose, xylitol, glycerol, acetate, and ethanol were analyzed by a high-performance liquid chromatography system (Merck Hitachi, Darmstadt, Germany), equipped with an Aminex HPX-87H column (Bio-Rad Hercules, USA) and a refractive index detector (L-7490, Merck Hitachi, Darmstadt, Germany). The column was eluted with 5 mM H_2_SO_4_ as mobile phase at a flow rate of 0.4 mL min^−1^, at 50 °C. Fermentation parameters [$Y_{{{\text{p}}/{\text{s}}}}^{\text{et}}$ (g g^−1^), ethanol yield; $Y_{{{\text{p}}/{\text{s}}}}^{\text{xy}}$ (g g^−1^), xylitol yield; *Y*_x/s_ (g g^−1^), biomass yield; *r*_s_ (g g_CDW_^−1^ h^−1^), specific d-xylose consumption rate, d-xylose consumption (%), *r*_et_ (g g_CDW_^−1^ h^−1^), specific ethanol productivity] were experimentally determined. Ethanol ($Y_{{{\text{p}}/{\text{s}}}}^{\text{et}}$, g g^−1^), xylitol ($Y_{{{\text{p}}/{\text{s}}}}^{\text{xy}}$ g g^−1^) and biomass (*Y*_x/s_, g g^−1^) yields were calculated by correlating Δ*P* produced (ethanol or xylitol) or Δ*X* produced (CDW) with Δ*S* consumed (d-xylose) at time of maximum ethanol production, respectively; specific ethanol productivity (*r*_et_, g g_CDW_^−1^ h^−1^) was determined during maximum volumetric ethanol production rate divided by the average of the biomass (CDW) in the same time interval; similarly, specific d-xylose consumption rate (*r*_s_, g g_CDW_^−1^ h^−1^) was calculated during maximum volumetric d-xylose consumption rate divided by the average of the biomass (CDW) in the same time interval, and d-xylose consumption (%) was determined as a percentage of initial d-xylose consumed at time of maximum ethanol production.

### Enzyme activities

For the enzymatic activity assay of XR and XDH in *Spathaspora* species, yeasts were grown in YPX medium as described above under both oxygen-limited conditions tested. After 16 h, cells were recovered, washed with sterile deionized water, and used to obtain crude cell-free extracts using Y-PER^®^ Yeast Protein Extraction Reagent (Pierce, Rockford, USA). Protein concentrations in the cell-free extract were determined by BCA Protein Assay Kit (Pierce, Rockford, USA). Enzymatic activities were determined by following the oxidation or reduction of the coenzymes at 340 nm using UV-2401 PC UV-PIS recording spectrophotometer (Shimadzu, Kyoto, Japan) at 25 °C, with an interval time of 1 s for recording and a total measuring time of 90 s for each reaction. Kinetic parameters of XR for xylose reduction were obtained in a reaction mixture containing 200 mM triethanolamine buffer pH 7.0, 10 mM NAD(P)H, 2 M d-xylose, cell-free extract and deionized water, while kinetic parameters of XDH for xylitol oxidation were obtained in a reaction mixture containing 200 mM glycine buffer pH 9.0, 500 mM MgCl_2_, 60 mM NAD(P)^+^, 2 M xylitol, cell-free extract, and deionized water. A value of 5.33 mM^−1^ cm^−1^ was used for the absorption coefficient of NAD(P)H. One unit was defined as the generation of 1 μmol NAD(P)H per min. The specific enzyme activities were given in units (U) per mg protein [39]. This experiment was performed in biological duplicate.

Enzymatic activities of XR in TMB strains (3501, 3502, 3503, and 3504) grown in aerobic conditions in shake flasks containing 2× YNB medium with 50 g L^−1^ xylose and 50 mM potassium hydrogen phthalate pH 5.5, at 30 °C and 200 rpm for 48 h, were also performed. Reaction mixtures and the calculations of specific enzyme activities were conducted as described above for determination of *Spathaspora* species XR activities.

### Genomic analysis of *XYL1* gene(s) in *Spathaspora* species

The genome regions of 15 Mbp containing *XYL1* gene(s) from *Spathaspora passalidarum* NRRL Y-27907^T^ (CBS 10155^T^) [29] and *Sp. arborariae* UFMG-CM-Y352^T^ (UFMG-HM-19.1A^T^, CBS 11463^T^) [30] were aligned and two sets of primers were designed (Additional file 3): one based on a consensus sequence covering *XYL1.1* of *Sp.passalidarum* and *XYL1* of *Sp. arborariae*, (SpspXYL1.1_F and SpspXYL1.1_R), previously assigned [29, 30]; and one covering *XYL1.2* of *Sp.passalidarum* (SppaXYL1.2_F and SppaXYL1.2_R), previously assigned in *Sp. passalidarum* [29]. The amplified fragments were submitted to DNA sequencing (STAB Vida, Portugal) to confirm *XYL1.1* gene sequence in *Sp. passalidarum* CBS 10155^T^, *XYL1* gene sequence in *Sp. arborariae* UFMG-CM-Y352^T^, and to identify *XYL1* genes sequences in the remaining strains. The sequences obtained were used to predict the amino acid residues forming the XR proteins and nucleotide and amino acid sequences were aligned by ClustalW multiple alignment using the free software BioEdit Sequence Alignment Editor (Ibis Biosciences). A phylogram of XYL1p was constructed with the software iTOL (EMBL, Germany).

### Transcript analysis of *XYL1.1* and *XYL1.2* expression in *Spathaspora passalidarum*

Samples from *Sp. passalidarum* cultures (CBS 10155^T^ and UFMG-CM-Y469) were taken for the transcript analysis of *XYL1.1* and *XYL1.2* along with samples for enzyme activities experiments (i.e., YPX medium, under moderate and severe oxygen-limited conditions, after 16 h). Samples were immediately frozen in liquid nitrogen, and stored at −80 °C prior to extraction. RNA extraction was performed with Direct-zol™ RNA MiniPrep w/TRI-Reagent^®^. First-Strand cDNA Synthesis was obtained with High-Capacity cDNA Reverse Transcription Kit (Applied Biosystems) with the following cycle: 25 °C 10 min, 37 °C 120 min, and 85 °C 5 min, in C1000 Touch™ Thermal Cycler (BIORAD). This step was performed in duplicate with real-time Power SYBR^®^ Green PCR (Applied Biosystems) with the following cycle: 94 °C 3 min, 40 cycles of 94, 55, and 72 °C, 0.5 min each temperature, and 72 °C 4 min. The mRNA expression levels of *SppaXYL1.1* and *SppaXYL1.2* were evaluated using actin (*SppaACT1*) and 18S ribosomal RNAs (*SppaRDN18*) as internal controls (primers list in Additional file 3), and analyzed by the Pfaffl method [40].

### Construction of *S. cerevisiae* TMB 3501, TMB 3502, TMB 3503, and TMB 3504

Plasmids and strains used for metabolic engineering of xylose fermentation in *S. cerevisiae* are listed in Table 5. *Escherichia coli* NEB 5-alpha (New England BioLabs, Ipswich, USA) used for cloning was grown at 37 °C on LB medium (tryptone, 10 g L^−1^; yeast extract, 5 g L^−1^; NaCl, 5 g L^−1^. *Saccharomyces cerevisiae* strains were grown at 30 °C on YPD medium (yeast extract, 10 g L^−1^; peptone, 20 g L^−1^; glucose, 20 g L^−1^) supplemented with 20 g L^−1^ agar whenever necessary. Plasmid DNA was prepared with GeneJET™ Plasmid Miniprep Kit (Thermo Scientific, Waltham, USA). Agarose gel DNA extraction was performed with QIAquick^®^ Gel Extraction Kit (Qiagen GmbH, Hilden, Germany). PCR amplification was conducted in C1000™ Thermal Cycler (Bio-Rad, Hercules, USA) using Phusion™ Hot Start High-Fidelity DNA Polymerase (Thermo Scientific, Waltham, USA) and dNTP from Thermo Scientific (Waltham, USA). PCR product purification was carried out with GeneJET™ PCR Purification Kit (Thermo Scientific, Waltham, USA). DNA sequencing was performed by Eurofins MWG Operon (Ebersberg, Germany) or by STAB Vida (Caparica, Portugal). Restriction endonucleases, Thermosensitive Alkaline Phosphatase, and T4 DNA Ligase from Thermo Scientific (Waltham, USA) were used for DNA manipulation. *Sp. passalidarum* strains CBS 10155^T^ and UFMG-CM-Y469 *XYL1.1* and *XYL1.2* genes were, respectively, amplified with the designed primers SppaXYL1.1_XbaIF, SppaXYL1.1_XbaIR, SppaXYL1.2_XbaIF, and SppaXYL1.2_XbaIR (Additional file 3). Purified amplicons were digested with *Xba*I, and the resulting fragments were inserted into the plasmid YIpOB8 [41] previously digested with *Xba*I to excise *Sc. stipitisXYL1* gene, creating YIpRC1, YIpRC2, YIpRC4, and YIpRC5. Correct orientation and sequence of the inserts were verified by restriction fragment analysis and sequencing. Competent *E. coli* NEB 5-alpha cells were transformed as described previously [42], and transformed *E. coli* strains were selected on LB plates containing 100 mg L^−1^ ampicillin. The constructed plasmids were purified and subsequently cleaved with *EcoRV* within the *URA3* gene and transformed into TMB 3044 [7] by the lithium acetate method [43]. Transformed yeast strains were selected on YNB plates (Yeast Nitrogen Base w/o amino acids, 6.7 g L^−1^; agar, 20 g L^−1^) containing 20 g L^−1^d-xylose.

**Table 5:** Yeast strains and plasmids constructed and/or used in this study

Strains and plasmids	Relevant features	References
Plasmids		
YpOB8	*URA3 TDH3p*-*XYL1*-*ADH1t, PGK1p*-*XYL2*-*PGK1t*	[41]
YpDR7	*URA3 TDH3p*-*XYL1(N272D)*-*ADH1t, PGK1p*-*XYL2*-*PGK1t*	[14]
YIpRC1	pOB8 *XYL1.1* *S. passalidarum* CBS 10155^T^	This work
YIpRC2	pOB8 *XYL1.1* *S. passalidarum* UFMG-CM-Y469	This work
YIpRC4	pOB8 *XYL1.2* *S. passalidarum* CBS 10155^T^	This work
YIpRC5	pOB8 *XYL1.2* *S. passalidarum* UFMG-CM-Y469	This work
*S. cerevisiae* strains		
TMB 3044	CEN.PK 2-1C, *MAT*a, *ura3*-*52*, Δ*gre3*, *his3::HIS3 PGK1p*-*XKS1*-*PGK1t*, *TAL1::PGK1p*-*TAL1*-*PGK1t, TKL1::PGK1p*-*TKL1*-*PGK1t*, *RKI1::PGK1p*-*RKI1*-*PGK1t, RPE1::PGK1p*-*RPE1*-*PGK1t*	[7]
TMB 3422	TMB 3044, *ura3*::YIpDR7	[14]
TMB 3501	TMB 3044, *ura3*:: YIpRC1	This work
TMB 3502	TMB 3044, *ura3*:: YIpRC2	This work
TMB 3503	TMB 3044, *ura3*:: YIpRC4	This work
TMB 3504	TMB 3044, *ura3*:: YIpRC5	This work

### Xylose fermentations under anaerobic conditions with recombinant *S. cerevisiae*

Anaerobic batch fermentation was carried out in a flat-bottomed 1.4-l Multifors bioreactor vessel (Infors AG, Bottmingen, Switzerland) with a working volume of 800 mL. Cells were pre-cultivated at 30 °C and 180 rpm in 250 mL shake flasks containing 50 mL of 2× YNB medium (Yeast Nitrogen Base w/o amino acids, 13.4 g L^−1^) with 50 g L^−1^d-xylose and 50 mM potassium hydrogen phthalate pH 5.5, recovered by centrifugation at 2600×*g* for 20 min, washed twice with sterile water, and inoculated into the bioreactor to a final concentration of 0.07 g_CDW_ L^−1^. Fermentation was conducted on 2 × YNB medium with 50 g L^−1^d-xylose, 0.01 g L^−1^ ergosterol and 0.5 mL L^−1^ antifoam RD emulsion (Dow Corning^®^, Midland, USA). Temperature was maintained at 30 °C, pH was controlled at 5.5 through addition of 3 M KOH, and stirring was set to 200 rpm. Anaerobic conditions were attained by sparging with nitrogen gas containing less than 5 ppm O_2_ (AGA GAS AB, Sundbyberg, Sweden) at a flow rate of 200 mL min^−1^ before inoculation. During fermentation, anaerobic conditions were maintained by the produced CO_2_ that diffused through a water lock. Cultures were sampled aseptically between 0 and 142 h and stored at −20 °C until analysis. Strain *S. cerevisiae* TMB 3422 [14], carrying *Sc. stipitisXYL1* (N272D), was used as reference for *S. cerevisiae* TMB 3504 carrying native *Sp. passalidarum* UFMG-CM-Y469 *XYL1.2*. The experiments were performed in biological duplicates. Growth was determined by measuring OD_620_ with a Ultrospec 2100 pro spectrophotometer (Amersham Biosciences, Uppsala, Sweden). Xylose, ethanol, xylitol, glycerol, and acetate were analyzed by high-performance liquid chromatography system (Waters, Milford, USA) with an Aminex HPX-87H ion exchange column (Bio-Rad Hercules, USA) and a refractive index detector (RID-6A, Shimadzu, Kyoto, Japan). The mobile phase was 5 mM H_2_SO_4_, at a flow rate of 0.6 ml, 45 °C. Cell dry weight was determined in triplicate by filtering a known volume of culture broth through a pre-weighed 0.45 μm Supor^®^ 450 Membrane filters (Pall Corporation, Port Washington, USA), washing with distilled water, drying in a microwave oven, and weighting. Fermentation parameters were calculated as described above.

## Additional files

10.1186/s13068-016-0570-6 Time course of substrate consumption (xylose at an initial concentration of 40–50 g L^−1^) and product formation by *Spathaspora* species under moderate and severe oxygen-limited batch fermentations.
10.1186/s13068-016-0570-6 ClustalW multiple alignment of XYL1p, XYL1.1p, and XYL1.2p amino acid sequences from *Spathaspora* species and *Scheffersomyces stipitis* (XYL1p N272D).
10.1186/s13068-016-0570-6 List and sequence of primers used in this study.

## Abbreviations

CDW: cell dry weight
OD: optical density
PCR: polymerase chain reaction
XR: xylose reductase
XDH: xylitol dehydrogenase
YNB: yeast nitrogen base
YPX: yeast extract peptone xylose
