# Supplementary material for: Exploring xylose metabolism in Spathaspora species: XYL1.2 from Spathaspora passalidarum as the key for efficient anaerobic xylose fermentation in metabolic engineered Saccharomyces cerevisiae
Source: Biotechnol Biofuels. 2016 Aug 5;9:167. doi: 10.1186/s13068-016-0570-6 (PMC4974763; doi:10.1186/s13068-016-0570-6)
Supplement: Supplementary file 1 — 10.1186/s13068-016-0570-6 Time course of substrate consumption (xylose at an initial concentration of 40–50 g L−1) and product formation by Spathaspora species under moderate and severe oxygen-limited batch fermentations. [file 13068_2016_570_MOESM1_ESM.pdf]

Additional file 1.pdf

Time course of substrate consumption (xylose at an initial concentration of 40-50 g.L^{-1}) and product formation by *Spathaspora* species under moderate (left column) and severe (right column) oxygen-limited batch fermentations. Symbols: square (continuous line) – xylose; circle (dashed line) – ethanol; triangle (continuous line) – xylitol; diamond (dashed line) – cells.

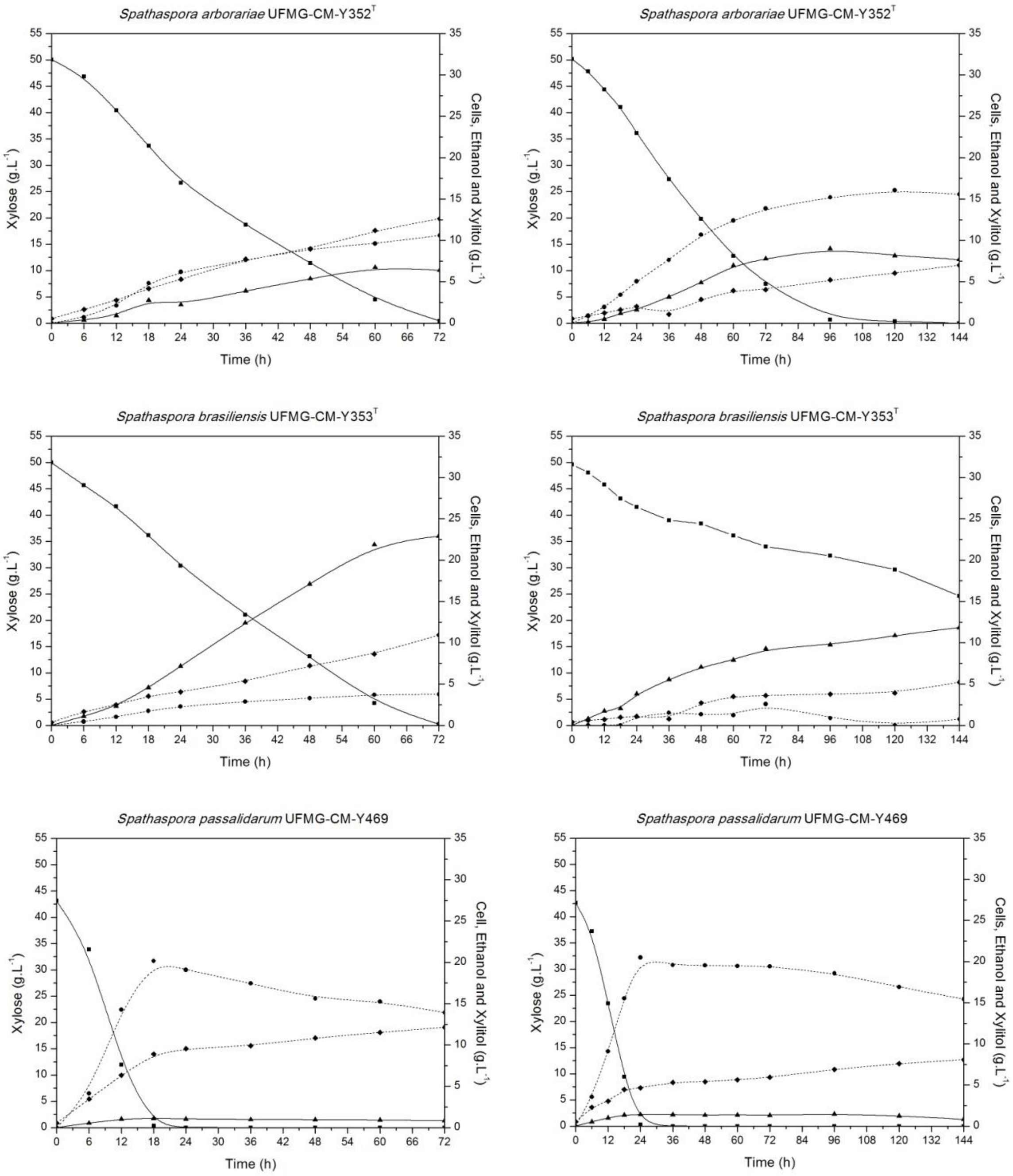

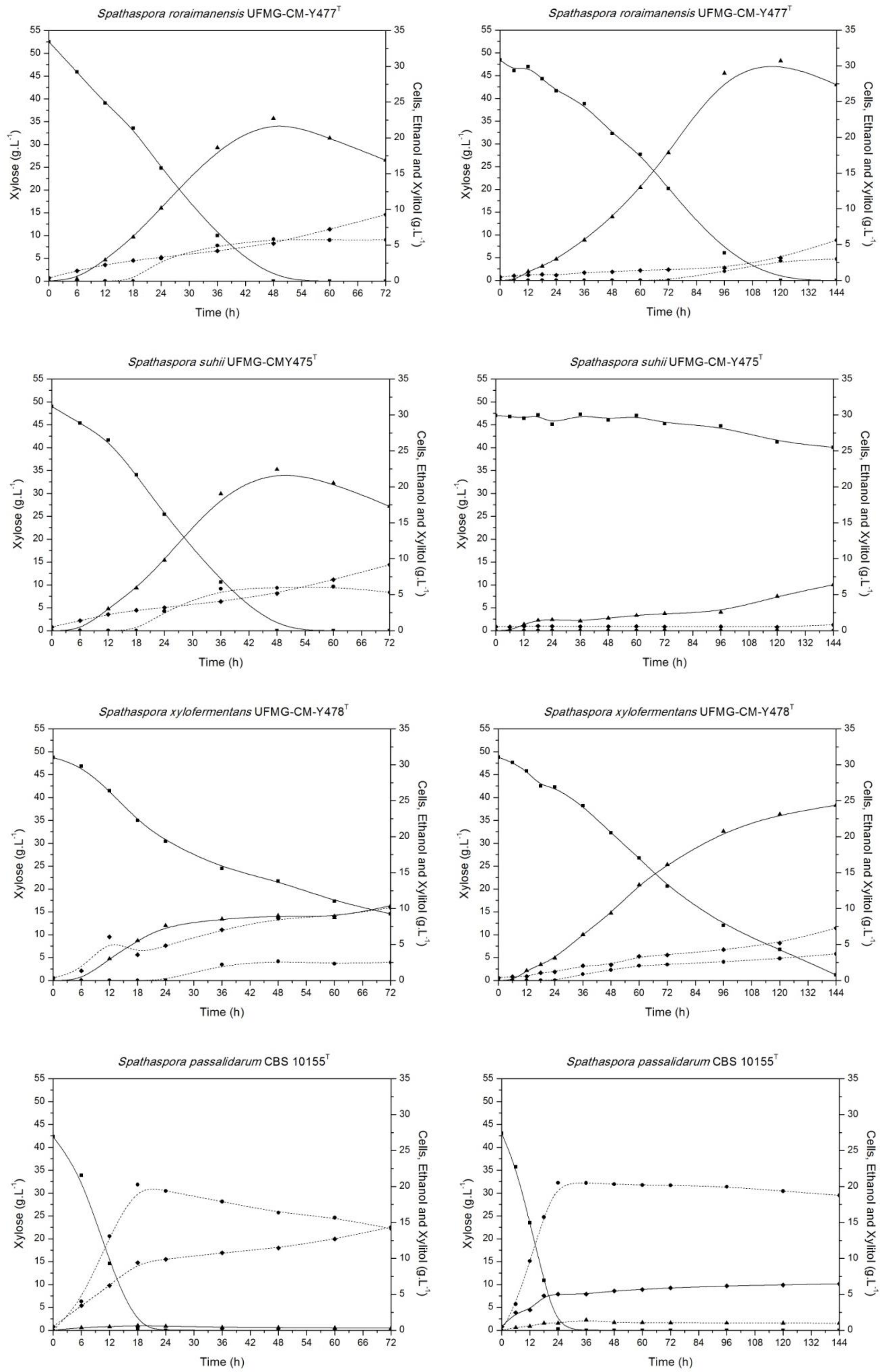
Spathaspora roraimanensis UFMG-CM-Y477T
Spathaspora roraimanensis UFMG-CM-Y477T
Spathaspora suhii UFMG-CMY475T
Spathaspora suhii UFMG-CM-Y475T
Spathaspora xylofermentans UFMG-CM-Y478T
Spathaspora xylofermentans UFMG-CM-Y478T
Spathaspora passalidarum CBS 10155T
Spathaspora passalidarum CBS 10155T
Xylose (g.L-1)
Cells, Ethanol and Xylitol (g.L-1)
Time (h)
