# Supplementary material for: Exploring xylose metabolism in Spathaspora species: XYL1.2 from Spathaspora passalidarum as the key for efficient anaerobic xylose fermentation in metabolic engineered Saccharomyces cerevisiae
Source: Biotechnol Biofuels. 2016 Aug 5;9:167. doi: 10.1186/s13068-016-0570-6 (PMC4974763; doi:10.1186/s13068-016-0570-6)

Additional file 2.pdf

ClustalW multiple alignment of XYL1p, XYL1.1p and XYL1.2p amino acid sequences from *Spathaspora* species and *Scheffersomyces stipitis* (XYL1p N272D). The highly conserved motif Ile-Pro-Lys-Ser (reported as the coenzyme binding site) is shown inside a rectangle box. The position site 271 (XYL1.2p) or 272 (XYL1p and XYL1.1p) is indicated by an arrow.

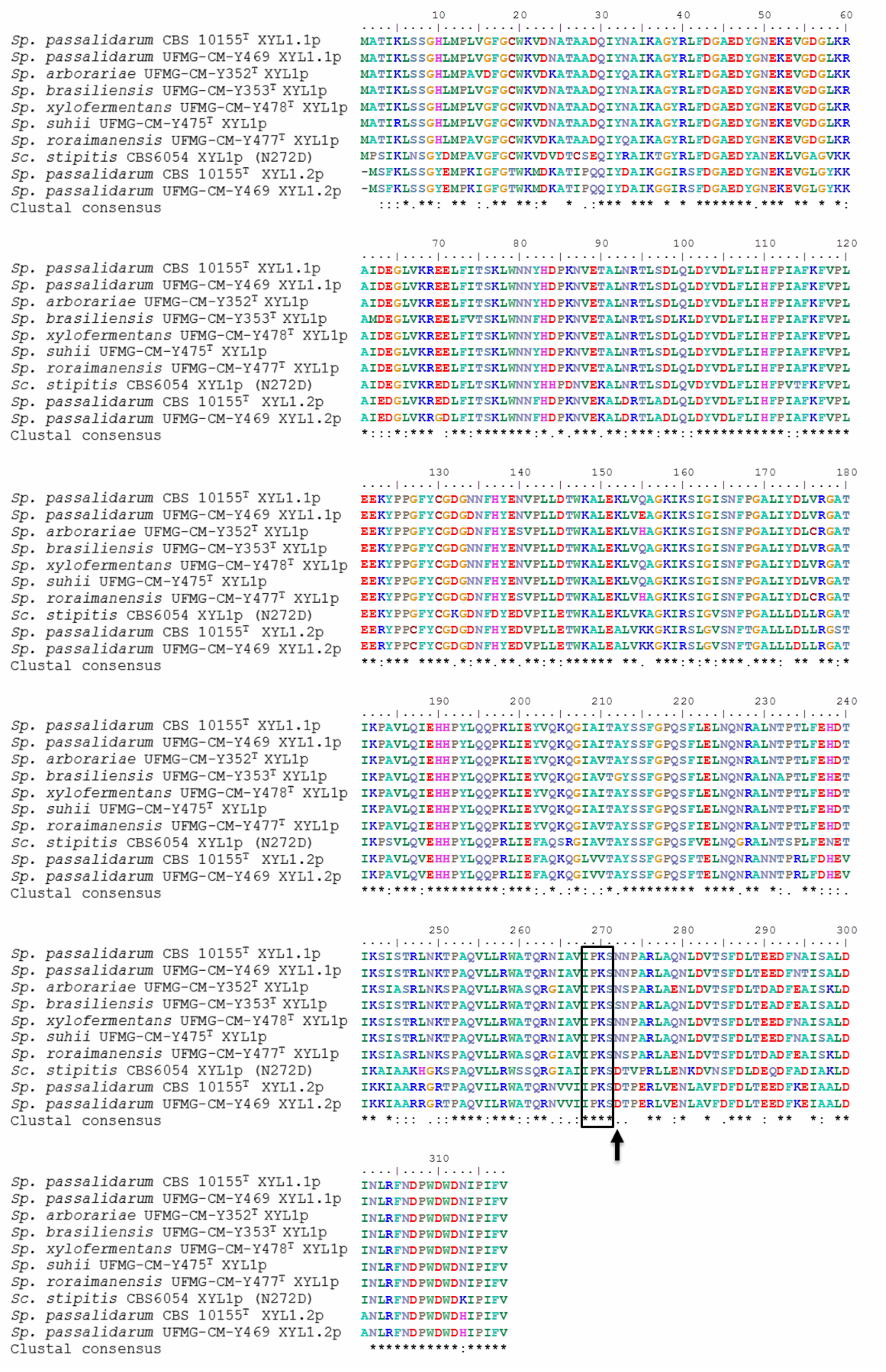

Supplement: Supplementary file 2 — 10.1186/s13068-016-0570-6 ClustalW multiple alignment of XYL1p, XYL1.1p, and XYL1.2p amino acid sequences from Spathaspora species and Scheffersomyces stipitis (XYL1p N272D). [file 13068_2016_570_MOESM2_ESM.pdf]
