# Supplementary material for: Exploring xylose metabolism in Spathaspora species: XYL1.2 from Spathaspora passalidarum as the key for efficient anaerobic xylose fermentation in metabolic engineered Saccharomyces cerevisiae
Source: Biotechnol Biofuels. 2016 Aug 5;9:167. doi: 10.1186/s13068-016-0570-6 (PMC4974763; doi:10.1186/s13068-016-0570-6)
Supplement: Supplementary file 3 — 10.1186/s13068-016-0570-6 List and sequence of primers used in this study. [file 13068_2016_570_MOESM3_ESM.pdf]

**Additional file 3.pdf**

List and sequence of primers used in this study.

| Primer | Sequence (5'-3') |
|---|---|
| SpspXYL1.1_F | TTTTGTGTAATCTCGGAT |
| SpspXYL1.1_R | TTTTATTAATGCTAATCGATAAC |
| SppaXYL1.2_F | ATTACTACTTACAAGTAAAACACA |
| SppaXYL1.2_R | ATTATCAGTGATCCAAAGAATGAA |
| SppaXYL1.1_XbaIF | GAC**TCTAGA**ATGGCTACTATTAAATT |
| SppaXYL1.1_XbaIR | GAC**TCTAGA**TTAAACGAAGATTGGAATG |
| SppaXYL1.2_XbaIF | GAC**TCTAGA**ATGTCTTTTAAATTATCT |
| SppaXYL1.2_XbaIR | GAC**TCTAGA**TTAAACAAAGATTGGAAT |
| Sppa_xyl1.1_rtFor | CTCAGGTCACTTGATGCCTTTAG |
| Sppa_xyl1.1_rtRev | TCTTTAAACCGTCACCGACTTCC |
| Sppa_xyl1.2_rtFor | GCCACCATTCCTCAGCAAATTTAC |
| Sppa_xyl1.2_rtRev | CTCTTAACAAGACCGTCTTCAATAGC |
| Sppa_act1_rtFor | AGATACCCAATTGAACACGGTATCG |
| Sppa_act1_rtRev | GATTTAGGATTCATTGGAGCTTCAG |
| Sppa_rdn18_rtFor | TCACCAGGTCCAGACACAATAAG |
| Sppa_rdn18_rtRev | GGTTAAGGTCTCGTTCGTTATCG |

Sites for restriction endonucleases are indicated in **bold**
